# Advanced Nanoindentation Testing for Studying Strain-Rate Sensitivity and Activation Volume

**DOI:** 10.1007/s11837-017-2536-y

**Published:** 2017-08-28

**Authors:** Verena Maier-Kiener, Karsten Durst

**Affiliations:** 1 Department Physical Metallurgy and Materials Testing, Chair Physical Metallurgy and Metallic Materials, Montanuniversität Leoben, Roseggerstr. 12, 8700 Leoben, Austria; 2 Physical Metallurgy, Technical University Darmstadt, Alarich-Weiss-Str. 2, 64287 Darmstadt, Germany

## Abstract

Nanoindentation became a versatile tool for testing local mechanical properties beyond hardness and modulus. By adapting standard nanoindentation test methods, simple protocols capable of probing thermally activated deformation processes can be accomplished. Abrupt strain-rate changes within one indentation allow determining the strain-rate dependency of hardness at various indentation depths. For probing lower strain-rates and excluding thermal drift influences, long-term creep experiments can be performed by using the dynamic contact stiffness for determining the true contact area. From both procedures hardness and strain-rate, and consequently strain-rate sensitivity and activation volume can be reliably deducted within one indentation, permitting information on the locally acting thermally activated deformation mechanism. This review will first discuss various testing protocols including possible challenges and improvements. Second, it will focus on different examples showing the direct influence of crystal structure and/or microstructure on the underlying deformation behavior in pure and highly alloyed material systems.

## Introduction

The strain-rate sensitivity exponent and the activation volume provide a fingerprint of the rate controlling mechanisms during thermally activated deformation. Over many years, nanoindentation has been used for assessing local mechanical properties such as hardness and modulus. To shed light into temperature and rate dependent deformation behavior, indentation creep tests were suggested,^1^ and Mayo and Nix^2^ reported superplastic deformation behavior of Pb and Sn during indentation. Especially for ultrafine-grained (UFG) and nanocrystalline (NC) materials, nanoindentation became a frequently used technique to determine strain-rate sensitivity (SRS). Schwaiger et al.^3^ published in 2003 a systematic comparison of nanoindentation and tensile testing on NC-Ni, where both techniques found a strongly grain size dependent SRS. Within the last decade, many studies on different materials classes, microstructures and environmental influences were reported in literature. Due to the limited amount of required material, especially the severe plastic deformation (SPD)^4^ and thin film^5^,^6^ communities used nanoindentation to gain information on the microstructure dependent deformation behavior of many face-centered cubic (FCC),^7^–^16^ body-centered cubic (BCC),^17^–^21^ and some hexagonal closed packed (HCP)^22^–^25^ metals. Also the SRS of nanocomposites such as Cu-Nb,^26^ nanoporous Cu and Au,^27^,^28^ or nowadays high-entropy alloys (HEA)^29^,^30^ were successfully investigated by nanoindentation. Besides the successful determination of positive SRS, few studies report experiments and results of negative SRS.^31^,^32^ Moreover, many other investigations dealing with various materials systems, such as glasses^32^–^34^ or intermetallic phases,^35^ can also be found.

The general analyses of SRS probed by nanoindentation is based on the conventional methods for macroscopic materials testing.^36^,^37^ For more detailed information, the reader is referred to classic literature such as the textbook by Caillard.^38^ Generally, the parameter quantifying the SRS of a material is *m*. It can be directly calculated from a simple power-law relationship between plastic stress $\sigma$ and applied strain-rate $\dot{\varepsilon }$ ($\sigma = \dot{\varepsilon }^{m}$). For nanoindentation experiments, correspondingly the indentation stress, thus the hardness *H*, is used:

1 $$ m = \frac{\partial \ln \sigma }{{\partial \ln \dot{\varepsilon }}}\sim \frac{\partial \ln H}{{\partial \ln \dot{\varepsilon }}} = \frac{{\partial \ln H_{2} - \partial \ln H_{1} }}{{\partial \ln \dot{\varepsilon }_{2} - \partial \ln \dot{\varepsilon }_{1} }} $$

The stress is directly connected to the hardness by a constraint factor ($C^{*}$), which generally depends on the elastic–plastic deformation behavior of the material^39^,^40^ ($\sigma = H/C^{*}$). Nevertheless, since only differences in stress are required to evaluate *m*, the exact choice of $C^{*}$ is only of minor concern. Furthermore, the activation volume $V^{*}$, which describes the plastic volume that is involved during the deformation, was described by Gibbs^41^,^42^ and is given for the macroscopic, uniaxial case as:

2 $$ v^{*} = kT \cdot \frac{{\partial \ln \dot{\varepsilon }}}{\partial \sigma } $$

with *k* Boltzmann constant, *T* testing temperature. For the local, multiaxial condition, it has to be converted or, as derived by Wei,^43^ *m* can be included:

3 $$ v^{*} = C^{*} \times \sqrt 3 \times kT \times \frac{{\partial \ln \dot{\varepsilon }}}{\partial H}\sim C^{*} \times \sqrt 3 \times \frac{kT}{m \cdot H} $$

Many macroscopic studies regarding thermally activated deformation processes can be found in the literature.^43^–^45^ Moreover, especially the comparison between local and global behavior was intensively used to get a better understanding of the acting deformation mechanism in UFG-materials.^46^,^47^ Nevertheless, one major aspect concerning all these studies are some experimental issues occurring during long-term nanoindentation, mainly caused by insuperably and omnipresent thermal drift.

In the following, two independent advanced nanoindentation methodologies for a reliable characterization of a strain-rate sensitive deformation behavior will be discussed on the example of a highly deformed austenitic steel (A220). Furthermore, several examples will demonstrate the significance of microstructure, crystal structure, and solute content on the time dependent deformation behavior and the underlying thermally activated deformation processes.

All shown indentation data and protocols were carried out using a standard platform Nanoindenter G200 (Keysight Tec, San Jose, USA). The instrument was equipped with a Berkovich diamond tip and a dynamic indentation unit (continuous stiffness measurement—CSM), where a harmonic oscillation is applied and the phase angles, the load, and the displacement amplitude together with the Sneddons equation^48^ are used to analyze the dynamic contact stiffness. This allows the determination of modulus and hardness continuously over indentation depth. For further information on that techniques and possible issues, the reader is referred to the literature.^49^–^51^ At this point, it should be mentioned that all presented approaches are not limited to this manufacturer but can be readily implemented on all available indentation systems equipped with a dynamic indentation unit.

## Experimental Possibilities and Challenges: Development of Advanced Testing Techniques

The basic principle for probing thermally activated deformation is to analyze the time dependent material response during a stress- or strain-rate controlled experiment, i.e., during strain-rate changes, stress relaxation, or creep loading. In nanoindentation, several testing possibilities are available, such as constant strain-rate,^52^ indentation creep,^53^ or indentation relaxation^54^–^56^ experiments. In all cases, the indentation strain-rate is the key parameter for a successful analysis. In the following discussion, several examples with possible issues are addressed, focusing mainly on pyramidal indenter tips (constant indentation strain, dependent on opening angle^39^) and only touching spherical indenters.

### Overview of Various Testing Options

The simplest but overall most frequently used indentation method is the constant load rate (cLR) indentation, where the indentation is conducted with a constant loading rate to a preset load, mostly without using any dynamic indentation technique. Therefore, loading times, load levels, and number of loading cycles can be adjusted by the user. Since in 1999 when Lucas and Oliver^52^ successfully showed that a constant indentation strain-rate can be accomplished by a proportional loading protocol, it has been obvious that the applied indentation strain-rate during each indentation segment is not constant. According to Lucas and Oliver, the indentation strain-rate can be described as follows (with *h* indentation depth, *P* indentation load, and *H* indentation hardness):

4 $$ \dot{\varepsilon } = \frac{{\dot{h}}}{h} = \frac{1}{2}\left( {\frac{{\dot{P}}}{P} - \frac{{\dot{H}}}{H}} \right) \approx \frac{1}{2}\frac{{\dot{P}}}{P} $$

Thus, for a constant strain-rate (cSR) experiment with pyramidal indenter tip geometry, a proportional loading protocol according to Eq. 4 is applied. This is commonly used in combination with a dynamic indentation unit, which analyzes, based on the dynamic stiffness and Sneddons equation, the mechanical data continuously as a function of indentation depth. Generally, the cSR is often used in the literature, but it is also known that macroscopic and local data were prone to differ in between,^9^ mainly caused by thermal drift effects. As shown in Refs. 32 and 57, cSR indentations with a strain-rate of 0.001 s^−1^ might easily end up with individual indentation times of more than 2 h, while a cSR of 0.05 s^−1^ leads to a total time of ~200 s. Taking into account low thermal drift values of 0.05 nm/s, this might easily sum up in displacement drift of more than 350 nm for low cSRs.

Applying Eq. 4 to cLR indentations shows^32^ that during conventional protocols with several partial loading–unloading cycles, constant loading times, and variable load levels, the strain-rate at the last point of each loading segment generally varies. A constant strain-rate is only achieved if the loading time to the different load levels remains constant during the partial loading segments, moreover, fully unloading the indenter tip after each loading segment. The corresponding contact stiffness and thus the mechanical properties will be evaluated after a holding segment at a constant peak load from the elastic unloading curve.^58^ This hold segment, however, still leads to some minor changes between the cSR and cLR methods due to strain-rate sensitive deformation behavior as recently pointed out by Leitner et al.^51^

### Nanoindentation Strain-Rate Jump Tests

To overcome these issues, Maier et al.^57^ and Alkorta et al.^19^ came up with an advanced indentation protocol, where abrupt strain-rate changes are applied during one single indentation segment.^57^ These kind of test are state-of-the-art in many macroscopic compression or tension tests, and they were successfully adjusted to the small-scale testing world. Within nanoindentation strain-rate jump tests, the applied strain-rate/indentation depth protocol can be adjusted individually, allowing variable indentation depths for the abrupt changes and different indentation strain-rates. Every parameter can be chosen to the requirement of the application and material.^32^ Additionally, several drawbacks of the cSR-method can be overcome with this one indentation method. For example, the total indentation time is significantly reduced because the initial indentation depth is performed at large strain-rates and segments with lower indentation strain-rates are performed at larger depths, effectively reducing testing times. Moreover, strain-rate jump tests also allow probing the mechanical properties at one single location, thus, e.g., the determination of SRS of individual phases or microstructural heterogeneities.^57^ For stable microstructural conditions, the evaluated strain-rate sensitivity is independent of the individual applied sequences of strain-rates and indentations depths. Nevertheless, in the case of unstable microstructures or during varying plastic strain conditions, the experimental sequence might slightly influence the evaluated materials properties. Therefore, the SRS should be always discussed as a function of hardness, since the rate sensitivity, the microstrcutural length scales and the hardness are closely related to each other.

**Fig. 1:**
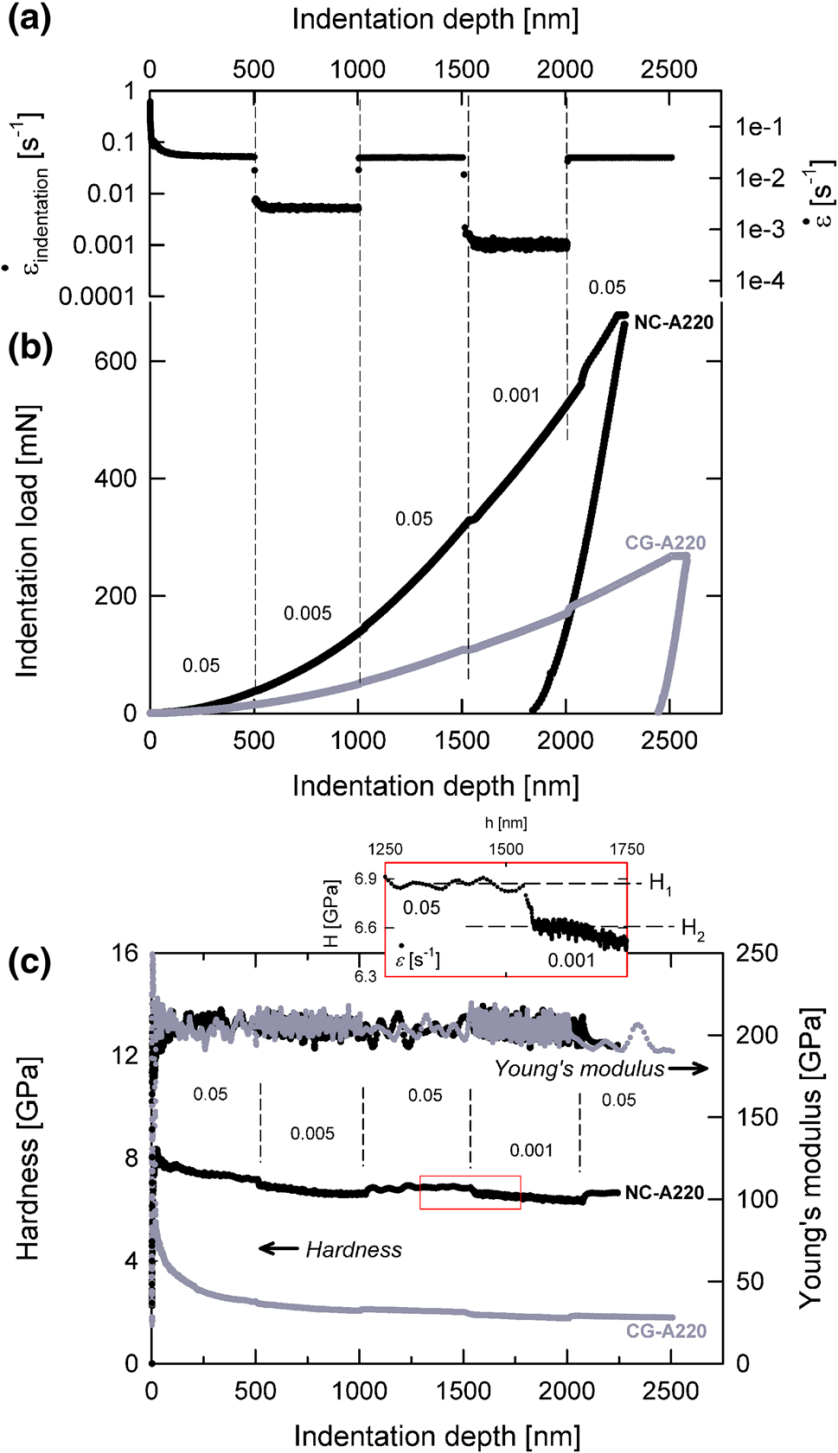
Nanoindentation strain-rate jump tests demonstrated on CG- and NC-A220 (austenitic steel), (a) preset indentation strain-rate protocol with four independent abrupt strain-rate changes, (b) corresponding load–displacement curves, and (c) hardness and Young’s modulus continuously over indentation depth (inlay presents a magnification of the third strain-rate jump for visualization of instantaneous SRS determination)

In Fig. 1, the applied indentation protocol of a nanoindentation strain-rate jump test (Fig. 1a) in an austenitic steel A220 is shown together with the corresponding load displacement curves (Fig. 1b) for a coarse-grained (CG) and a highly deformed NC microstructure. For both CG- and NC-conditions, a significant dependency of the load–displacement data on the applied strain-rate is found, with a larger effect on the NC-condition. After each SR-jump, the curve goes through a transient region adjusting correspondingly to the new applied SR. Figure 1c shows the calculated Young’s modulus and hardness data. The Young’s modulus is independent of the applied SR, only showing more data points in the regions with the lower indentation strain-rates and thus longer testing times. After each SR-jump, the hardness of the NC-condition goes through a transient region until a new SR-dependent, but depth-independent hardness is reached. Thereby, the hardness values of the first, third, and fifth strain-rate segment (0.05 s^−1^) exhibit about the same hardness values, indicating that no indentation size effect or microstructure changes are present.

To calculate a strain-rate sensitivity *m* or the corresponding activation volume $V^{*}$, the hardness difference according to the applied strain-rate changes has to be determined, as shown in the inset of Fig. 1c. These analysis have to be performed in both a global manner (average values cSR regions^57^) for materials showing a depth-independent hardness and/or instantaneous (e.g., in the presence of an ISE^18^) manner for materials with any kind of indentation size effect as the size effect would affect the global evaluation of *m*.

### Nanoindentation Long-Term Creep Tests

Indentation creep or relaxation testing is another common methodology to probe thermally activated processes.^54^,^59^,^60^ In these tests, the indentation tip is pushed into the material to a preset indentation load or displacement, and afterwards, the load is held constant for a preset creep time. To assess the creep properties, the change in displacement has to be recorded over a long time range.

The topic is highly discussed but at the same time frequently used, and several analysis of interpretation can be found in literature.^59^,^61^–^67^

Some main methodical aspects, especially while comparing global and local data, have to be pointed out and kept in mind:

- During macroscopic creep testing, a constant stress level is applied and a change in creep strain and strain-rate are recorded. Pyramidal indentation tips imply, however, a constant representative strain to the material, which varies in the plastic zone but is independent of indentation depth. During constant load creep, a change in the indentation hardness is found and both applied stress and strain-rates are changing. This makes a comparison with macroscopic constant stress creep tests rather complicated.^65^ Furthermore, during uniaxial creep testing, a steady-state secondary creep regime is analyzed, which cannot be reached with pyramidal long-term indentations.^32^,^63^
- The use of spherical indentation tips during creep^67^ probes a varying indentations strain with changing indentation depth due to the not self-similarity of the sphere, which comes closer to macroscopic creep regimes but still suffers from the not uniform stress–strain fields underneath the indenter.^63^
- Long time creep experiments can suffer from thermal drift issues; therefore, some groups only apply creep experiments of less than a minute.^68^ Nevertheless, there the initial setting processes in the plastic zone are hardly related to long time creep mechanisms.

To investigate the environmental influences during nanoindentation creep testing at room temperature, Maier et al.^34^ performed two individual constant load indentations for 2 h at four different indentation depths on the reference material fused quartz. They found that the displacement signal for all indentations showed some significant scatter that obviously could not be explained with actual mechanical properties. Further long-time indentations of 24 h on FQ^32^ could prove that there is a relation between indentation temperature evolution and recorded displacement data.

These thermal drift related issues have already been well known for a long time, although they are still ignored in many works. In 1992, Weihs and Pethica^69^ came up with a first dynamically corrected long-term creep method. This method was later further improved by Pethica and Asif^70^ and Goldsby et al.^71^ All groups used a correction technique based on the dynamic contact stiffness *S* during both the loading and the constant load segment. This contact stiffness is less prone to thermal drift influences than the recorded indentation displacement. While the displacement is recorded far away from the indented area in the gauge of the indenter head, the dynamically recorded contact stiffness gives knowledge about the true apparent contact at a frequency of several Hertz during the experiment. Together with Sneddon’s equation,^48^ this allows the determination of the true contact area *A*_c_:

5 $$ S = \frac{2\beta }{\sqrt \pi } \times E_{\text{R}} \times \sqrt {A_{\text{c}} } = S_{\text{CSM}} $$

6 $$ A_{\text{c}} = \frac{\pi }{{4\beta^{2} }} \times \frac{{S^{2} }}{{E_{\text{R}}^{2} }} $$

As described in Ref. 10 in more detail, from the know contact area, the drift corrected contact depth *h*_c_ can be evaluated. Together with the Oliver–Pharr equation $h = h_{\text{c}} + \varepsilon \cdot \frac{P}{{S_{\text{CSM}} }}$, drift issues can be taken into account for both displacement *h* and contact depth *h*_c_. Finally, according to Joslin and Oliver,^72^ this corrected contact area *A*_c_ can be also used to determine the hardness, simply based on contact stiffness and a known modulus:

7 $$ H_{\text{corrected}} = \frac{P}{{A_{\text{c}} }} = \frac{{4\beta^{2} }}{\pi }E_{\text{R}}^{2} \cdot \frac{P}{{S^{2} }} $$

**Fig. 2:**
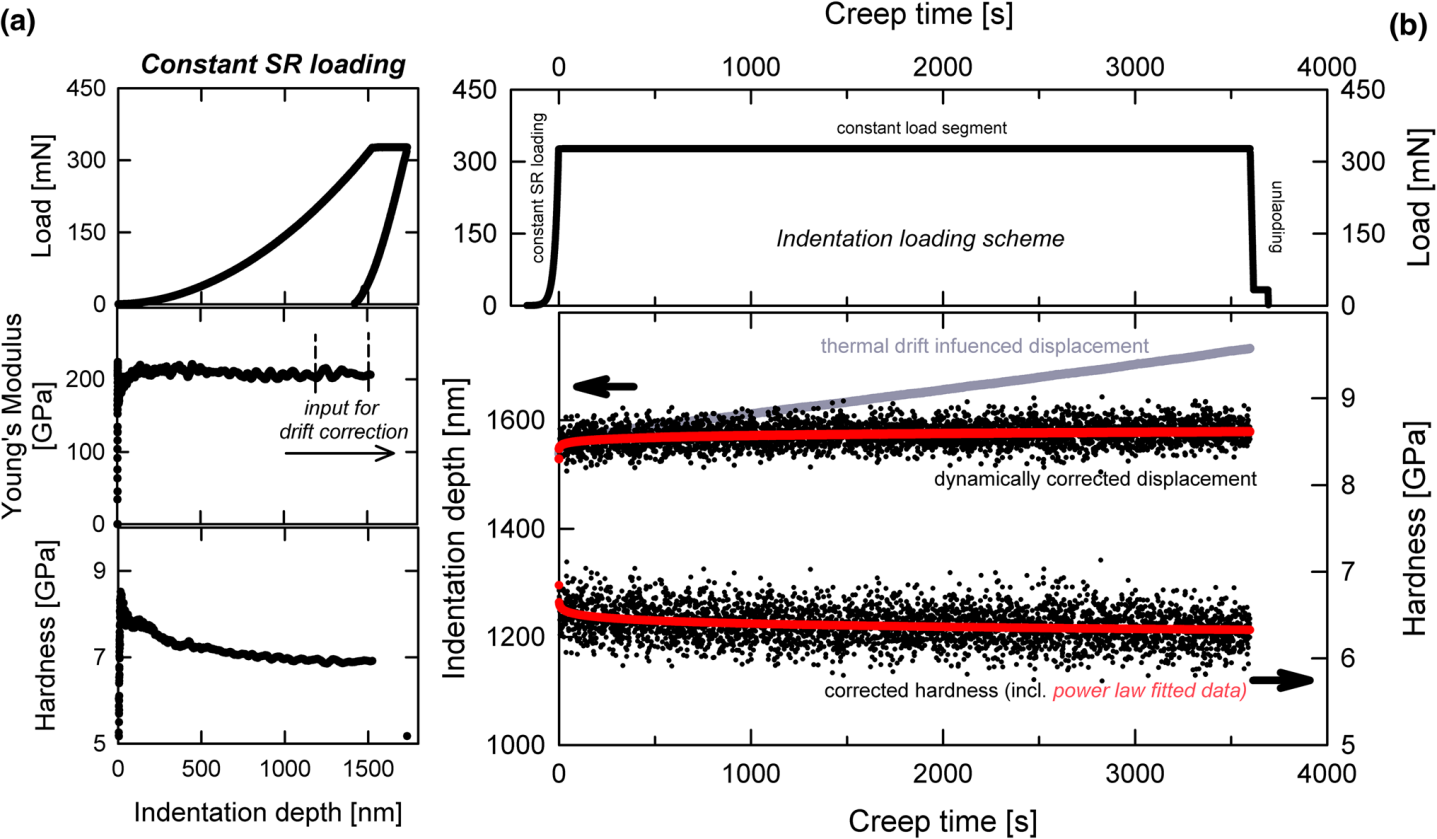
Nanoindentation long-term creep experiments on HPT-deformed austenitic steel A220, with dynamically corrected displacement signal, (a) cSR (0.05 s^−1^) loading segment to a preset indentation depth of 1500 nm (load–displacement, Young’s modulus and hardness) and (b) indentation creep protocol (load versus indentation time) and corresponding creep displacement with original drift influenced (gray), dynamically corrected (black), and fitted data (red) (Color figure online)

A long time creep indentation experiment is shown in Fig. 2 on A220. Figure 2a shows the mechanical data of the cSR loading segment, including load–displacement behavior, hardness, and modulus. The reduced modulus *E*_r_ from the loading segment serves at the same time as basis for the dynamic correction,^10^ as shown in Eqs. 5 and 6.

After reaching the preset indentation depth, the raw load is held constant at that level for a preset time (Fig. 2b). The recorded displacement data as well as the corrected data according to the method suggested by Weihs and Pethica^69^ are plotted in the lower part. Although the thermal drift was less than 0.05 nm/s, the recorded original displacement data differs significantly to the corrected one. The corrected hardness and displacement exhibit some scatter, which is due to the use of the square of the dynamic contact signal. Any noise in the dynamic stiffness is thereby amplified. Using a power law function to fit the data, this can be circumvented and the resulting values can be treated analytically. Further analysis concerns the determination of equivalent creep/strain-rates $\dot{\varepsilon }_{\text{equivalent}} = \frac{{\dot{h}}}{h}$ from the corrected indentation signal or a transformation of the hardness into an equivalent stress $\sigma_{\text{equivalent}} = \frac{{H_{\text{corrected}} }}{{C^{*} }}$.

Applying this dynamic correction protocol to several data sets shows extensively that the mentioned differences in displacement signal just collapse to one reproducible curve, as demonstrated in Ref. 34.

A further successful application for this method is the investigation of SRS of thin films, where the thickness of the investigated film is too low for other kind of tests.^5^ Additionally, for high-temperature testing, especially for systems with an apparent thermal mismatch due to a nonheated indentation tip, this method might act as a possibility to overcome the drastic issues of thermal drift during indentation.^10^ But also for indentation systems with an independently heated tip and sample, a reduction of thermal drift issues especially during the indentation times was demonstrated for ufg-Au.^34^

### Other Small-Scale Testing Techniques and Expanding to Harsh Environmental Conditions

Since nanoindentation testing is a common tool to probe mechanical properties and is not limited anymore to ambient conditions, several methods and protocols can also be used for high-temperature testing. Results of both nanoindentation strain-rate jump tests^8^,^17^,^35^,^68^ as well as nanoindentation creep tests^26^–^28^,^34^ are reported over a wide range of temperatures and strain-rates, shedding more light on local thermally activated deformation processes. High temperature testing, however, leads to further challenges regarding thermal stability and testing procedures.^73^

The effect of different strain regimes on thermally activated deformation processes can be addressed by spherical indentation. Spherical tips have no self-similar geometry; thus, the implied strain varies with indentation stress and contact radius. Recently, Feldner et al.^74^ implemented strain-rate jump tests with a spherical tip. Localized creep tests with spherical tips are also reported in the literature.^75^ Spherical indenter tips have thereby many advantages but also one drawback, which is the required spherical shape, which is only easily achieved for diamond indenters. Nevertheless, at this point, it is worth mentioning that especially the calibration of the nonperfect, spherical tip shapes, the controlling parameters of cSR indentation experiments, as well as the correct analysis of the data are still in an emerging state.

Besides nanoindentation, which always represents a multiaxial loading situation, uniaxial micropillar compression experiments can also be used to examine thermally activated deformation parameters on a very local scale, using, for example, creep,^76^ jump,^73^ or stress relaxation tests.^77^ Wehrs et al.^12^,^78^ demonstrated that the different protocols for the same material lead to comparable results. Finally, also bulge test techniques were modified regarding a reliable testing of SRS.^79^

### Quantification of Thermally Activated Processes

**Fig. 3:**
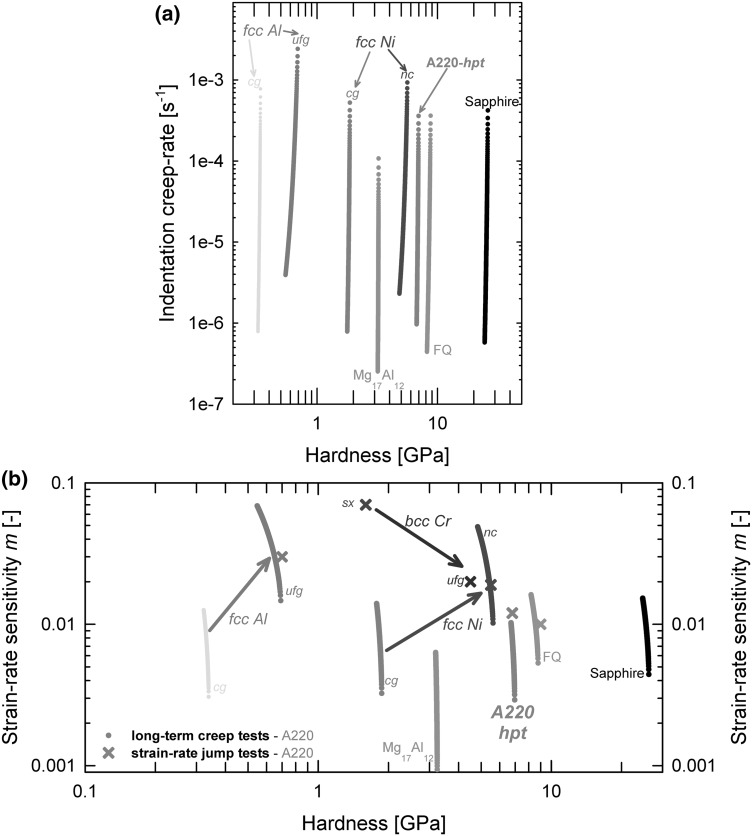
Analysis of nanoindentation long-term creep experiments. (a) Norton-plot for NC-A220 (data from Fig. 2) compared with different materials, crystal structures, and microstructures. (b) Corresponding SRS over hardness for various materials and testing protocols (creep results appear in continuous manner and strain-rate jump tests as crosses); FCC-metals: Al, Ni,^10^ BCC-metal: Cr,^17^ intermetallic phase: Mg_17_Al_12_, reference materials: FQ,^10^ Sapphire (some data are taken with permission by Cambridge Press^10^ and Elsevier^17^)

With a reproducible long-term creep and strain-rate jump test, the underlying thermally activated deformation mechanisms can be determined for a wide variety of materials. For the analysis, the *m* and $V^{*}$ are determined according to Eqs. 1 and 2. Correlating these material parameters, a unique characteristic for each material, depending on crystal structure and microstructure but also on temperature and deformation history, can be evaluated. Figure 3a shows a Norton plot for A220 and various other materials, where the calculated displacement creep rate is plotted over hardness during the constant load segment. Figure 3b displays the corresponding SRS exponent from creep tests and strain-rate jump tests for several fcc and bcc metals as well as a glass and a ceramic. Generally jump and creep tests are in good accordance with each other,^32^,^68^ and as described for macroscopic compression tests,^80^ the SRS depends on the hardness (which is for jump tests mostly neglected) since during creep experiments, *m* is given by the slope of the hardness versus equivalent strain-rate.

The mechanical properties shown in Fig. 3 spread over two orders of magnitude in hardness, five orders in strain-rate, and two orders in strain-rate sensitivity. Consequently, a variety of material behavior and underlying deformation mechanisms were tested. We will at this point not go into all details, but only summarize a few general findings. Reference materials such as FQ or Sapphire exhibit little SRS but are definitively not negligible, which should be kept in mind when using them as athermal standards. Mg_17_Al_12_ as a representative for an intermetallic phase shows at RT almost no thermal activation. Nevertheless, overcoming 150°C, it was reported that the deformation behavior suddenly changes within a narrow temperature interval to a highly SR-dependent behavior.^35^ For pure Cr as a BCC metal, underneath its critical temperature *T*_c_, the SRS increases with increasing grain size,^17^,^18^ approaching the single crystal condition due to the more dominant thermally activated movement of screw dislocations. The activation volume $V^{*}$, however, is rather independent of the microstructure as the kink-pair-mechanism prevails. In Al or Ni, which are common examples for pure FCC-metals, the SRS increases with grain refinement. Interestingly, comparing pure Ni with a smaller grain size of ~50 nm compared to ~300 nm for Al, the former exhibits a lower rate sensitivity. Moreover, the hpt-A220 has a grain size comparable to NC-Ni but lower values of SRS. This indicates that simple thermally activated dislocation annihilation at grain boundaries^46^ or grain boundary sliding^47^ is not sufficient to explain this behavior; rather, the interplay and detailed atomistic details of the mechanisms involved should be considered in the future. Initial studies on various materials and microstructures indicate that besides the dislocation-GB interaction, also solute-content affected grain boundary motions might play a dominant role, as focused in the next section.

## Thermally Activated Processes Depending on Crystal Structure, Microstructure, and Solute Solution Content

Since strain-rate sensitive deformation behaviors have manifold characteristics and also shed more light into the individual underlying deformation mechanism, in the following two subsections, interesting materials systems will be discussed in closer detail.

### Zr-Based Materials—Influence of Crystal Structure and Microstructure

Zr-based materials can be found in many different structures and microstructures. Zr is common as a base element in many BMGs, but also it is studied as a pure metal in the nanocrystalline condition and industrially used for example in cladding applications.

**Fig. 4:**
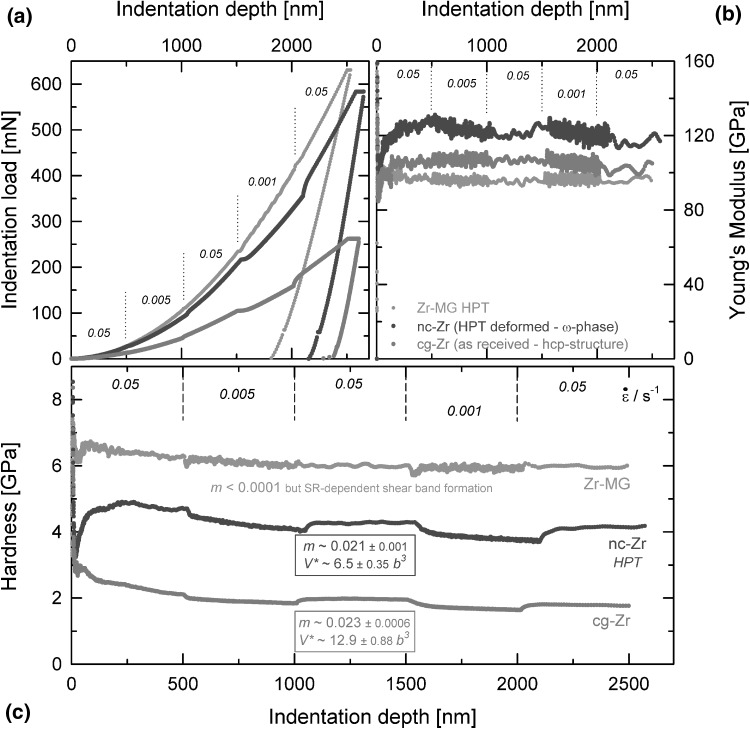
Zr-based materials (Zr-MG, NC-Zr, CG-Zr) tested by nanoindentation strain-rate jump tests (a) load–displacement behavior indicating SRS for all three microstructures, (b) crystal-structure dependent Young’s modulus, and (c) strain-rate dependent hardness

During nanoindentation of BMGs, it is known that the material shows strain-rate dependent shear band formation.^81^,^82^ This can be also seen in Fig. 4a for a Zr metallic glass (Zr-MG, for further details, see Ref. 83), where the load–displacement data show some serrated flow for low strain-rates. Next to that, the data of CG- and a highly deformed NC-condition are plotted. Corresponding Young’s modulus and hardness data are shown in Fig. 4b and c. While Zr-MG exhibits the highest hardness but is independent of strain-rate, the modulus is, as expected for BMGs, the lowest. CG-Zr has an hcp crystal structure, which is also prone to show some SRS in CG states due to the prismatic slip. This can be clearly seen in the hardness data, leading to a SRS of ~0.023 and a corresponding $V^{*}$ ~ 13·*b* ^3^. The transient behavior occurs smoothly, and the materials require about 100-nm indentation depth until a new steady deformation state is reached again. SPD leads to a further change in the crystal structure and to the formation of an ω-phase,^24^ which can be also seen by the changed Young’s modulus. The hardness significantly increases, but the SRS stays the same. The characteristic of the jump itself, however, has slightly changed to a more abrupt distinct change in hardness with less transients.

### Highly Alloyed Materials—High-Entropy Alloys (HEA)

**Fig. 5:**
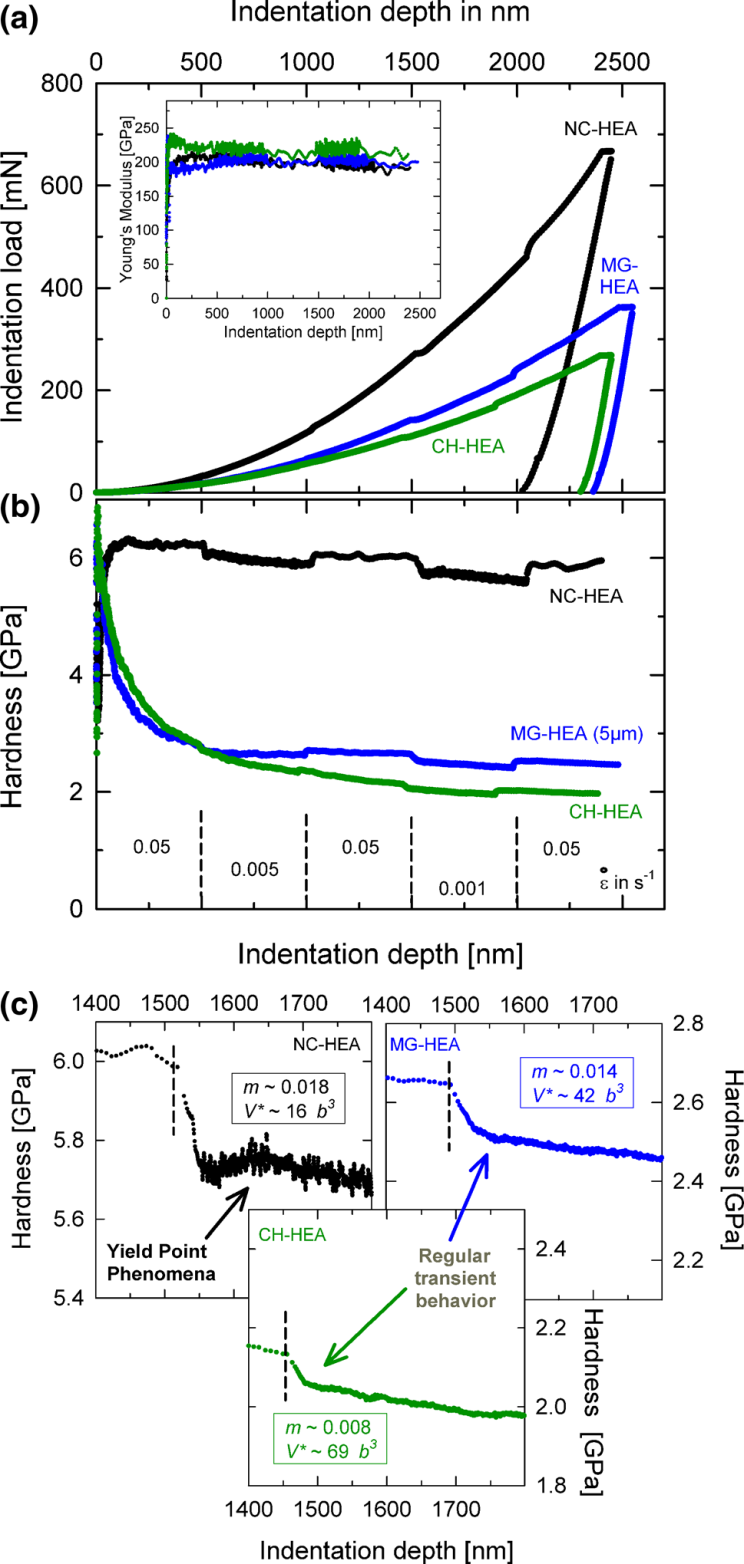
Influence of solid solution content and microstructure on SRS of an equiatomic HEA with different microstructures (according to Maier-Kiener et al.^29^ and reprinted with permission by Elsevier)

HEA are a further intensively discussed materials class. Besides their infinite number of compositions and their unique mechanical properties, these HEAs exhibit an interesting behavior regarding their thermal activation.^30^,^84^,^85^ As already reported for macroscopic tests,^86^ it was also shown during nanoindentation of the CoCrMnNiFe-HEA that the single crystalline like CH-condition exhibits a surprisingly high SRS of around 0.01 for a single crystalline FCC metal (Fig. 5c). A refinement of the microstructure by HPT leads to a significant increase in hardness and to an increasing SRS. Corresponding behavior is seen in the activation volume, where for all states, surprisingly low values were calculated. According to these results, it can be concluded that in these highly alloyed systems, a further mechanism in the single crystalline state might act since conventional pure fcc-metals are expected to be strain-rate insensitive with corresponding activation volumes around ~1000·*b* ^3^.

Furthermore, with grain refinement, also the jump characteristics change from a pronounced transient behavior to a so-called yield point phenomena, where the hardness first decreases followed by a slight increase and a further decrease. This is also found in other highly alloyed, NC-materials (such as NC-A220—Fig. 1c) and was also reported previously by Van Petegem et al.^87^ for NC-Ni-Fe alloys during transient tension testing. Nevertheless, the overall underlying deformation behavior is not fully understood yet and subject to ongoing investigations.

## Conclusion and Outlook

Nanoindentation is a versatile tool for probing thermally activated processes on a local scale. Simple methodological adjustments lead to reliable testing results paired with a minimization of testing time and a reduction of number of indentations. This opens further possibilities regarding a detailed investigation of the properties of individual phases, heterogeneous microstructures, and thin coatings. In this application oriented review, different examples were presented to demonstrate how SRS and activation volume can be employed for a fundamental understanding of governing deformation mechanisms but also how alloying and solute content as well as microstructure influence the thermally activated deformation behavior, in terms of both the individual load–displacement behavior as well as the overall hardness evaluation.

Generally it is stated that:

- Distinct testing protocols have to be carefully selected regarding reliability, application, and expected gain.
- Strain-rate jump tests allow the calculation of *m* and $V^{*}$ in a limited amount of time based on a single indentation.
- Dynamically corrected long-term creep experiments can be used to probe low strain-rate regions, but the achieved strain-rates depend on the SRS of the material itself.
- Crystal structure, microstructure, and testing temperature significantly influence the deformation behavior.
- The absolute values of strain-rate sensitivity *m* and activation volume $V^{*}$ can be directly used to identify the underlying rate-limiting deformation mechanism. Moreover, the quantitative changes upon, for example, different material modifications, loading rates, or temperatures are indicative of the interaction, competition, and change of different contributing plasticity mechanisms.

Nonetheless, for an overall scale bridging and comprehensive understanding of thermally activated processes, macroscopic and microstructural investigations are additionally recommended to allow for a reliable investigation over several length scales.
